# Human Milk Oligosaccharide LNnT Attenuates Colonic Barrier Dysfunction and Associated Cognitive Impairment via Modulating Sphingolipid Metabolism and Gut Microbiota

**DOI:** 10.3390/molecules31091410

**Published:** 2026-04-24

**Authors:** Minghui Wang, Liuying Zhu, Jinqiang Liao, Lulu Bao, Hongyan Li, Zeyuan Deng, Jing Li, Liufeng Zheng, Bing Zhang

**Affiliations:** 1 State Key Laboratory of Food Science and Resources, Nanchang University, Nanchang 330047, China; 407900230126@email.ncu.edu.cn (M.W.); 407900220121@email.ncu.edu.cn (J.L.); bll00301@163.com (L.B.); lihongyan@ncu.edu.cn (H.L.); dengzy@ncu.edu.cn (Z.D.); lijing66@ncu.edu.cn (J.L.); zhenglf2018@ncu.edu.cn (L.Z.); 2 Ningbo Municipal Hospital of Traditional Chinese Medicine (TCM), Affiliated Hospital of Zhejiang Chinese Medical University, Ningbo 315010, China; zly756315763@163.com; 3 International Institute of Food Innovation, Nanchang University, Nanchang 330051, China

**Keywords:** Lacto-N-neotetraose, intestinal protection, sphingolipid metabolism, intestinal microbiota

## Abstract

This study focuses on Lacto-N-neotetraose (LNnT), a core component of human milk oligosaccharides. Although LNnT has been demonstrated to promote early intestinal development and maintain gut homeostasis, its protective mechanism against D-galactose-induced intestinal injury and associated cognitive impairment remains unclear. This investigation systematically examined the protective effects and underlying mechanisms of LNnT against D-gal-induced colonic damage and cognitive impairment in mice. The results demonstrated that LNnT not only significantly improved systemic physiological phenotypes and upregulated the expression of colonic tight junction proteins to repair the intestinal barrier, but also effectively enhanced learning and memory abilities in mice. Concurrently, LNnT reduced serum proinflammatory factor levels, elevated the anti-inflammatory factor IL-10, and alleviated oxidative stress. Furthermore, LNnT remodeled the gut microbiome structure by increasing microbial diversity, enhancing beneficial bacteria abundance, and promoting short-chain fatty acid production. Untargeted metabolomics analysis further revealed that LNnT corrected metabolic disturbances by regulating key sphingolipid molecules (ceramide, sphingosine, S1P) and the expression of related metabolic enzymes (ACER2, SphK2). In summary, this study suggests that LNnT mitigates intestinal injury and improves cognitive function, potentially through modulation of the gut microbiota–sphingolipid metabolism axis, although further causal validation is warranted. These findings provide a mechanistic foundation for future studies exploring its potential as a functional dietary ingredient.

## 1. Introduction

The intestinal barrier serves as the critical interface separating the body’s internal environment from the contents of the intestinal lumen. The structural and functional integrity of this barrier plays an essential role in maintaining immune homeostasis and overall health [1]. In recent years, growing evidence from animal and human studies indicates that intestinal homeostasis, particularly mucosal integrity, is one of the core factors influencing overall health and disease states [2,3,4]. As a core component of the host defense system, the intestinal barrier constitutes a sophisticated and dynamic equilibrium involving multiple protective layers: the mechanical barrier (intestinal epithelial cells and their tight junctions), the chemical barrier (mucus, digestive secretions), the immune barrier (gut-associated lymphoid tissue), and the biological barrier (gut microbiota) [5]. Among these, intestinal epithelial tight junction proteins (e.g., Claudins, Occludin, ZO-1) form the molecular foundation for maintaining the selective permeability of the mechanical barrier. Impairment of these proteins leads to abnormal increases in intestinal permeability, known as “leaky gut”, allowing undigested food antigens, pathogens, and endotoxins (e.g., lipopolysaccharides) to translocate into the circulatory system [6]. This heightened intestinal permeability is now understood to be a critical contributing factor and a key amplifier in the pathogenesis of chronic systemic inflammation, establishing a complex and often bidirectional relationship with the pathophysiology of multiple chronic diseases including inflammatory bowel disease, metabolic syndrome, non-alcoholic fatty liver disease, and neuropsychiatric disorders [7,8,9,10].

Chronic low-grade inflammation represents a core pathophysiological mechanism driving the multi-system dysfunctions described above. When the intestinal barrier is compromised, persistent entry of substances like endotoxins into the circulation activates pattern recognition receptors on innate immune cells (e.g., macrophages). This leads to sustained activation of pro-inflammatory signaling pathways such as the nuclear factor-Κb (NF-κB) pathway, triggering an inflammatory state characterized by elevated levels of pro-inflammatory factors including tumor necrosis factor-α (TNF-α), interleukin-1β (IL-1β), and interleukin-6 (IL-6) [11]. Studies have shown that the inflammatory environment can further compromise intestinal tight junction structures, potentially creating a vicious cycle that involves elevated oxidative stress levels and contributes to exacerbated tissue damage [12,13]. To investigate the interaction between chronic inflammation and intestinal barrier injury and to explore potential intervention strategies, establishing a stable and reliable animal model is crucial. The D-galactose (D-gal)-induced subacute injury model is a widely utilized tool in this field. Its core mechanism lies in the fact that prolonged excessive D-gal metabolism in vivo leads to the accumulation of advanced glycation end products (AGEs), mitochondrial dysfunction, and redox imbalance, thereby effectively simulating chronic oxidative stress and inflammatory states [14,15]. The D-gal model is primarily regarded as an accelerated aging model driven by oxidative stress and AGE accumulation, rather than a direct recapitulation of natural aging or human disease pathogenesis [14,16]. Its underlying mechanisms may differ from the multifactorial etiologies of age-related intestinal barrier dysfunction or neurodegenerative disorders in humans [17]. Natural aging results from decades of interplay among cumulative environmental exposures, genetic predisposition, and chronic low-grade inflammation (“inflammaging”), whereas the D-gal model induces a relatively acute, chemically-driven oxidative insult [18]. Thus, although this model effectively recapitulates specific oxidative and inflammatory phenotypes, its capacity to fully replicate the complex, progressive pathology of gut barrier failure observed in conditions such as inflammatory bowel disease or age-related leaky gut syndrome remains limited. Despite these limitations, the D-gal model remains a valuable tool for dissecting specific mechanistic pathways associated with oxidative stress- and inflammation-driven pathologies. Its principal advantage lies in providing a controlled and reproducible system for evaluating the efficacy of interventions targeting these core pathological processes [14]. Notably, Wang demonstrated that D-gal administration in tree shrews induced cognitive impairment concomitant with reduced intestinal occludin expression and decreased gut microbial diversity, establishing a direct link between peripheral intestinal alterations and central nervous system dysfunction [19]. Similarly, Yan reported that ginsenoside compound K alleviated D-gal-induced mild cognitive impairment by modulating gut microbiota-mediated short-chain fatty acid metabolism and restoring intestinal barrier integrity [20]. These findings collectively support the feasibility of employing the D-gal model to investigate gut–brain axis dysfunction. Accordingly, the present study utilizes this model to explore the protective effects of LNnT against oxidative stress- and inflammation-induced intestinal barrier injury and associated cognitive impairment.

Human milk oligosaccharides (HMOs) are a natural and complex mixture of carbohydrates present in human milk, ranking as the third most abundant solid component following lactose and lipids [21]. Evidence from preclinical and infant studies has increasingly suggested that HMOs possess the capacity to modulate immune responses, combat pathogenic infections, reduce the risk of allergies, and confer potential benefits for cognitive development [22,23,24,25,26,27]. Of greater significance, HMOs can directly or indirectly enhance gastrointestinal barrier function, maintain gut microbial homeostasis, and attenuate inflammatory responses, thereby promoting intestinal health in infants, children, and even adults [28,29,30]. It should be noted, however, that most of the current evidence regarding the physiological roles of HMOs has been obtained from infant or neonatal models. Consequently, the translation of these findings to adult physiology remains constrained, and further studies are needed to validate their efficacy in broader populations. Clinically, HMOs have been demonstrated to reduce the incidence of necrotizing enterocolitis (NEC) in preterm infants [31]. Emerging evidence further suggests that specific HMOs, such as 2′-fucosyllactose (2′-FL), may ameliorate metabolic disorders by restoring the balance of gut microbiota and innate immune homeostasis [32,33].

In recent years, numerous studies have suggested that LNnT, a primary neutral core component of HMOs, can modulate the composition of the gut microbiota [29,34]. Notably, growing evidence indicates that the composition of the gut microbiota is not only related to intestinal health but is also involved in regulating brain physiological functions via the gut–brain axis [35,36]. Nevertheless, the precise mechanisms underlying this bidirectional communication remain incompletely characterized and warrant further investigation. Recent studies have reported that disrupting the gut microbiota in colitis mice significantly interferes with gut–brain communication, thereby inducing neuroinflammation and brain pathological changes [37]. These findings highlight the gut microbiota as an important mediator of gut–brain signaling. Consequently, targeting the modulation of gut microbiota composition, as LNnT is known to do, and their metabolites represents a promising strategy for preventing or treating diseases associated with imbalances in the gut microbiota (dysbiosis). Moreover, impairment of intestinal barrier integrity can facilitate the translocation of inflammatory molecules and harmful bacterial byproducts from the intestinal lumen into systemic circulation. These substances can then reach the brain, directly triggering neuroinflammatory signaling cascades [38,39]. Our previous work has demonstrated that early-life supplementation with LNnT promotes intestinal development, enhances intestinal barrier function, and beneficially modulates the structure of the gut microbiota by fostering the growth of beneficial bacteria while inhibiting harmful ones [29]. However, whether LNnT can improve D-gal-induced intestinal barrier dysfunction and associated cognitive impairment through regulation of the gut microbiota, as well as the underlying mechanisms involved, remains to be fully elucidated.

Therefore, this study aims to investigate the potential protective effects of LNnT against D-gal-induced colonic injury and associated cognitive impairment, and to explore the underlying mechanisms. Employing a multi-omics integration strategy, we comprehensively assessed physiological phenotypes, colonic barrier integrity, systemic inflammation, oxidative stress levels, and learning and behavioral indices. Further integrating 16S rRNA gene sequencing with untargeted metabolomics, we focused on validating the following core hypothesis: LNnT improves intestinal barrier function by regulating the gut microbiota–sphingolipid metabolism axis, thereby exerting its gut–brain axis protective effects. Furthermore, the effects of LNnT were compared with those of the common prebiotic galacto-oligosaccharides (GOS) to evaluate its functional specificity. This study provides novel experimental evidence and mechanistic insights into the physiological functions of LNnT, laying a crucial theoretical foundation for developing dietary components that alleviate chronic inflammation, intestinal barrier damage, and associated cognitive decline.

## 2. Results

### 2.1. Effect of LNnT Intervention on Relevant Phenotypes in D-Gal-Induced Mice

**Figure 1:**
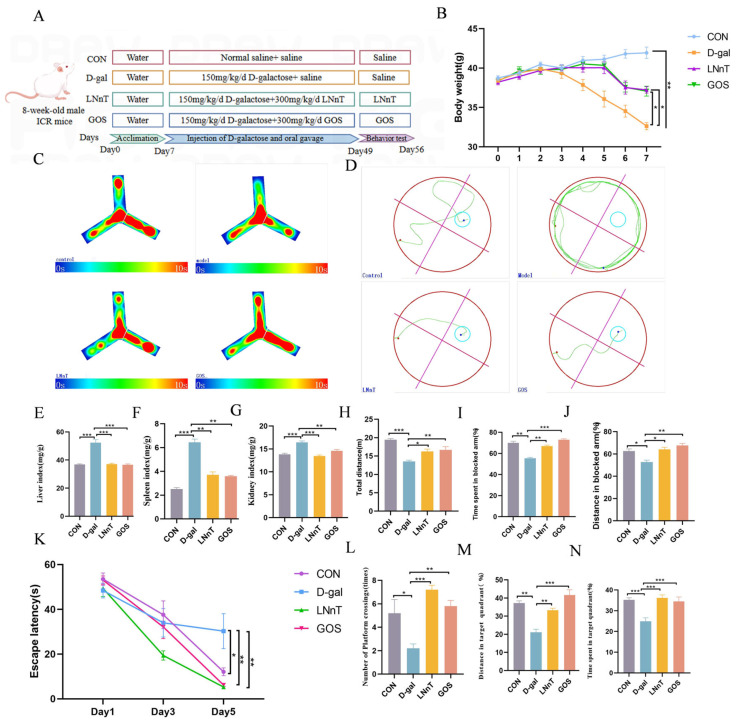
Effects of LNnT or GOS intervention on body weight, organ indices, and cognitive function in D-gal-induced mice. (**A**) Experimental design diagram. (**B**) Body weight changes over the 7-week experimental period. (**C**) Representative movement heatmaps in the Y-maze test. (**D**) Representative swimming trajectories in the Morris water maze (MWM) probe trial on day 5. (**E**) Liver index. (**F**) Spleen index. (**G**) Kidney index. (**H**) Distance in blocked arm (%). (**I**) Total distance in the Y-maze. (**J**) Time spent in the blocked arm (%). (**K**) Escape latency. (**L**) Number of platform crossings. (**M**) Proportion of distance traveled in the target quadrant (%). (**N**) Proportion of time spent in the target quadrant (%). All data are presented as the mean ± SEM (*n* = 8). * *p* < 0.05, ** *p* < 0.01, *** *p* < 0.001 vs. D-gal group using one-way ANOVA followed by Duncan’s multiple range test.

The animal experimental protocol is depicted in in Figure 1A. Body weight was monitored weekly throughout the study, with results presented in Figure 1B. Mice in the D-gal group exhibited a significant weight reduction starting from the third week compared to the control group, whereas the LNnT and GOS treatment groups showed a gradual decline beginning in the fifth week. By the end of the seventh week, the control group reached a body weight of 41.0 ± 0.7 g, while the D-gal group displayed a significantly lower weight (32.62 ± 0.5 g). In contrast, the LNnT and GOS groups maintained intermediate body weights (37.0 ± 0.5 g), with comparable temporal trends observed between the two intervention groups.

As shown in Figure 1E–G, organ indices for the liver, kidney, and spleen were significantly elevated in the D-gal group relative to the control group (*p* < 0.001). Notably, both LNnT and GOS interventions attenuated these increases, exhibiting significant differences from the D-gal group (*p* < 0.01), but no statistical distinction from the control group. The elevated organ indices observed in D-gal-treated mice may reflect a combination of processes including edema and inflammatory infiltration. These alterations reflect substantial organ tissue injury and associated functional deficits in the experimental model.

Spatial recognition and memory were evaluated using the Y-maze test, with representative movement heatmaps displayed in Figure 1C. Compared to the control group, mice in the D-gal group exhibited significant behavioral alterations in the Y-maze test. The total movement distance, which reflects general locomotor activity, was significantly reduced (*p* < 0.05, Figure 1I), suggesting potential changes in motor function. Meanwhile, the time spent in the novel arm (*p* < 0.01, Figure 1J) and the distance traveled in the novel arm (*p* < 0.05, Figure 1H), both indicators of spatial recognition memory, were also significantly decreased. Spontaneous alternation index, a classic measure of spatial working memory, was also assessed. As shown in Figure S1, the spontaneous alternation rate was significantly decreased in the D-gal group (19.44%) compared with the control group (32.35%) (*p* < 0.01). LNnT intervention significantly restored the spontaneous alternation rate to 34.26% (*p* < 0.01) relative to the D-gal group, whereas GOS treatment produced no significant improvement (26.50%, *p* > 0.05). Conversely, LNnT and GOS administration significantly improved these parameters (*p* < 0.05), with no notable differences between the two treatment groups. These findings suggest that LNnT alleviates D-gal-induced motor and cognitive dysfunction and further confirm that LNnT can ameliorate D-gal-induced spatial working memory impairment.

To further assess learning and memory capabilities, the Morris water maze (MWM) test was employed. During the five-day training phase, escape latency progressively decreased across all groups (Figure 1K). Compared to the control group, the D-gal group exhibited significantly prolonged escape latency (*p* < 0.05), indicating learning impairment, whereas LNnT and GOS treatments substantially shortened latency. In the probe trial on day 6 (conducted without the platform), the D-gal group showed fewer platform crossings and lower percentages of swimming distance and time in the target quadrant (Figure 1L–N). Importantly, LNnT and GOS interventions reversed these deficits, resulting in significantly improved spatial memory performance compared to the D-gal group (*p* < 0.01). To exclude the potential influence of motor function on cognitive performance, swimming speed and total swimming distance during the MWM test were analyzed. No significant differences were observed among the four groups (Figure S1), indicating that the observed changes in escape latency and probe trial performance were not confounded by alterations in locomotor activity. Together, these results demonstrate that LNnT can mitigate D-gal-induced impairments in spatial learning and memory.

### 2.2. Effect of LNnT Intervention on Colonic Morphology and Intestinal Barrier Function in D-Gal-Induced Mice

**Figure 2:**
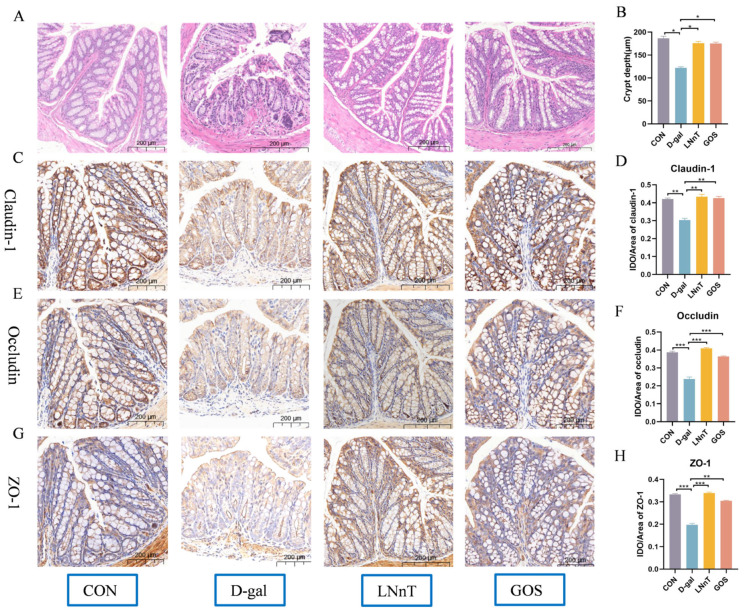
Effect of LNnT intervention on the morphology of mouse colon tissues and intestinal barrier proteins. (**A**) Representative pictures of H&E stained colon sections (magnification of 200×). (**B**) Colon crypt depth. (**C**) Representative images of Claudin-1’s colonic AB/PAS staining (magnification of 200×). (**E**) Representative images of Occludin’s colonic AB/PAS staining (magnification of 200×). (**G**) Representative images of ZO-1’s colonic AB/PAS staining (magnification of 200×). (**D**) Quantitative analysis of Claudin-1. (**F**) Occludin. (**H**) ZO-1 expression in colon tissue. All data were presented as means ± SEM (*n* = 3). * *p* < 0.05, ** *p* < 0.01, *** *p* < 0.001, vs. D-gal group using one-way ANOVA followed by Duncan’s multiple range test.

To evaluate D-gal-induced colonic damage, morphological changes in colon tissues were assessed via histological analysis. As shown in Figure 2A, the D-gal group exhibited significant pathological alterations, including disruption of the tissue architecture, partial loss of crypt structures in the mucosal layer, and a marked reduction in crypt depth (*p* < 0.05, Figure 2B). Notably, these degenerative changes were substantially ameliorated in mice receiving LNnT or GOS intervention.

Tight junction (TJ) proteins play a critical role in maintaining intestinal mucosal barrier integrity. Immunohistochemical staining was employed to quantify the expression of key TJ proteins (Claudin-1, Occludin, and ZO-1) in colonic tissues (Figure 2C–H). Compared with the control group, the D-gal group exhibited a significant reduction in the expression of Claudin-1, Occludin, and ZO-1 (*p* < 0.01), indicating impaired intestinal barrier function. In contrast, both LNnT and GOS interventions significantly upregulated the expression of these TJ proteins relative to the D-gal group (*p* < 0.01), thereby exerting a protective effect on intestinal barrier integrity.

These findings suggest that LNnT and GOS may hold therapeutic potential in ameliorating intestinal barrier dysfunction and attenuating associated inflammatory responses in D-gal-induced mice.

### 2.3. LNnT Attenuates D-Gal-Induced Oxidative Stress and Inflammation in Mice

**Figure 3:**
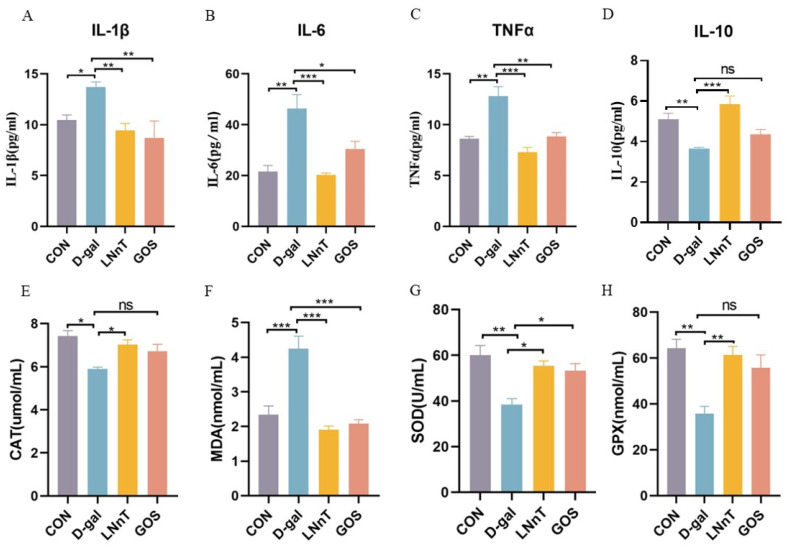
Effects of LNnT intervention on the inflammatory response in mouse serum. (**A**) The contents of IL-1β, (**B**) IL-6, (**C**) TNFα, and (**D**) IL-10 in the serum of each group; effects of LNnT on antioxidant level in the colon tissue of mice, (**E**) the contents of CAT, (**F**) MDA, (**G**) SOD, and (**H**) GPX in the colon tissue of each group. All data were presented as means ± SEM (*n* = 5). * *p* < 0.05, ** *p* < 0.01, *** *p* < 0.001 vs. D-gal group using one-way ANOVA followed by Duncan’s multiple range test. ns, not significant.

To assess systemic inflammatory status, serum concentrations of pro-inflammatory cytokines (IL-1β, IL-6, TNF-α) and the anti-inflammatory cytokine IL-10 were quantified via enzyme-linked immunosorbent assay (ELISA). As depicted in Figure 3A–D, compared with the control group, the D-gal group exhibited significantly elevated levels of IL-1β (*p* < 0.05), IL-6 (*p* < 0.01), and TNF-α, accompanied by a marked reduction in IL-10 (*p* < 0.05). Both LNnT and GOS interventions significantly decreased these pro-inflammatory cytokine levels (*p* < 0.05). Notably, LNnT treatment further significantly increased serum IL-10 levels (*p* < 0.001), whereas GOS failed to induce a statistically significant change in IL-10 compared to the D-gal group. These findings suggest that LNnT alleviates D-gal-induced systemic inflammation by modulating the balance between pro- and anti-inflammatory cytokines.

To evaluate oxidative stress, commercial assay kits were used to measure malondialdehyde (MDA) levels and the activities of antioxidant enzymes (catalase [CAT], superoxide dismutase [SOD], and glutathione peroxidase [GPx]) in D-gal-induced aging mice. As shown in Figure 3E–H, the D-gal group displayed significantly increased MDA levels (*p* < 0.05) and significantly decreased activities of CAT (*p* < 0.001), SOD (*p* < 0.01), and GPx (*p* < 0.01) compared to controls. In contrast, LNnT intervention effectively counteracted these changes, significantly reducing MDA levels (*p* < 0.001) and enhancing the activities of CAT (*p* < 0.05), GPx (*p* < 0.01), and SOD (*p* < 0.05). Although GOS also significantly reduced MDA levels (*p* < 0.001) and enhanced SOD activity (*p* < 0.05), its effects on CAT and GPx activities failed to reach statistical significance. These results indicate that LNnT exerts a potent antioxidative effect in D-gal-induced mice.

### 2.4. Effect of LNnT Intervention on Gut Microbiota in D-Gal-Induced Mice

**Figure 4:**
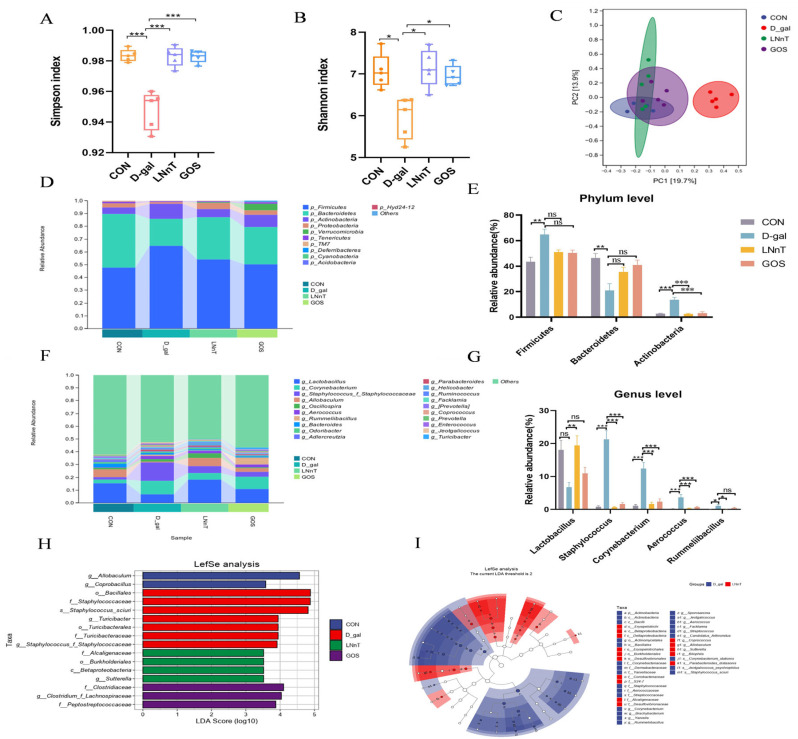
Effects of LNnT intervention on the diversity and structure of intestinal microbes. (**A**) Simpson Index. (**B**) Shannon index, (**C**) Analysis of β diversity among different groups by PCA, Supplementation of LNnT altered gut microbiota composition of mice injured by D-gal. Comparison of the representative taxonomic abundance of the four groups, at phylum level (**D**,**E**), and genus level (**F**,**G**). Effects of LNnT on intestinal flora in D-gal-injured mice. (**H**) Taxonomic cladogram obtained using LEfSe analysis. (**I**) Scores for the abundances of different taxa using linear discriminant analysis (LDA). All data were presented as means ± SEM (*n* = 5). * *p* < 0.05, ** *p* < 0.01, *** *p* < 0.001 vs. D-gal group using one-way ANOVA followed by Duncan’s multiple range test. ns, not significant.

The gut microbiota plays a critical role in maintaining intestinal barrier function, and its dysbiosis is recognized as a key driver of pathogenesis in various intestinal and systemic disorders. To characterize microbial community changes, 16S rRNA gene sequencing was performed on intestinal samples from each group, with microbial diversity assessed using the Simpson and Shannon indices. Compared with the control group, the D-gal group exhibited significantly reduced Simpson and Shannon indices (*p* < 0.05), indicating decreased microbial diversity. In contrast, both LNnT and GOS interventions significantly increased these alpha diversity indices (*p* < 0.05, Figure 4A,B). Venn diagram analysis revealed 410 shared operational taxonomic units (OTUs) across all four groups, with the control, D-gal, LNnT, and GOS groups harboring 1664, 524, 1717, and 1067 unique OTUs, respectively (Figure S2). Consistent with the observed changes in alpha diversity indices (Simpson and Shannon, Figure 4A,B), the D-gal group showed a marked reduction in unique OTU numbers, while LNnT and GOS interventions increased these numbers, suggesting that LNnT mitigates D-gal-induced alterations in gut microbial richness.

Beta diversity analysis, which reflects inter-group differences in community composition, was performed using principal coordinate analysis (PCoA) and non-metric multidimensional scaling (NMDS). PCoA revealed clear separation between the control and D-gal groups, whereas the LNnT and GOS groups clustered closely with the control group, indicating reversal of D-gal-induced microbial structural shifts (Figure 4C). Consistent results were obtained via NMDS (Figure S3).

At the phylum level, the dominant microbiota across all groups included *Firmicutes*, *Bacteroidetes*, *Actinobacteria*, and *Proteobacteria* (Figure 4D). Compared with the control group, the D-gal group exhibited a significant increase in the relative abundance of *Firmicutes* and *Actinobacteria*, accompanied by a decrease in *Bacteroidetes* (*p* < 0.01, Figure 4E). Compared with the D-gal group, both LNnT and GOS interventions exhibited a trend toward reduced Firmicutes abundance and increased Bacteroidetes abundance, while significantly decreasing Actinobacteria abundance (*p* < 0.001, Figure 4E).

At the genus level, the D-gal group showed significantly lower relative abundance of Lactobacillus (*p* < 0.01) and higher abundances of *Staphylococcus* (*p* < 0.001), *Corynebacterium* (*p* < 0.001), *Aerococcus* (*p* < 0.001), and *Rummeliibacillus* (*p* < 0.05) compared with the control group. LNnT and GOS interventions reversed these alterations by increasing Lactobacillus abundance and decreasing the abundances of *Staphylococcus*, *Corynebacterium*, *Aerococcus*, and *Rummeliibacillus* relative to the D-gal group, though the increase in Lactobacillus in the GOS group did not reach statistical significance (Figure 4F,G). These genera have been associated with various functional roles in the gut; for instance, Lactobacillus is often linked to gut health, whereas Staphylococcus includes species that can act as opportunistic pathogens depending on host context [40,41,42].

LEfSe was used to identify microbial taxa significantly enriched in each group (LDA score > 3.0, *p* < 0.05), resulting in 16 discriminative taxa. The control group was enriched with g_*Allobaculum* and g_*Coprobacillus*; the D-gal group was characterized by seven OTUs, including o_*Bacillales*, f_*Staphylococcaceae*, and s_*Staphylococcus sciuri* as the most influential; the LNnT group showed enrichment in f_*Alcaligenaceae*, o_*Burkholderiales*, c_*Betaproteobacteria*, and g_*Sutterella*; and the GOS group was associated with f_*Clostridiaceae*, g_*Clostridium*, and f_*Peptostreptococcaceae* (Figure 4H,I).

### 2.5. Effect of LNnT Intervention on Changes in Fecal SCFA Levels in D-Gal-Induced Mice

**Figure 5:**
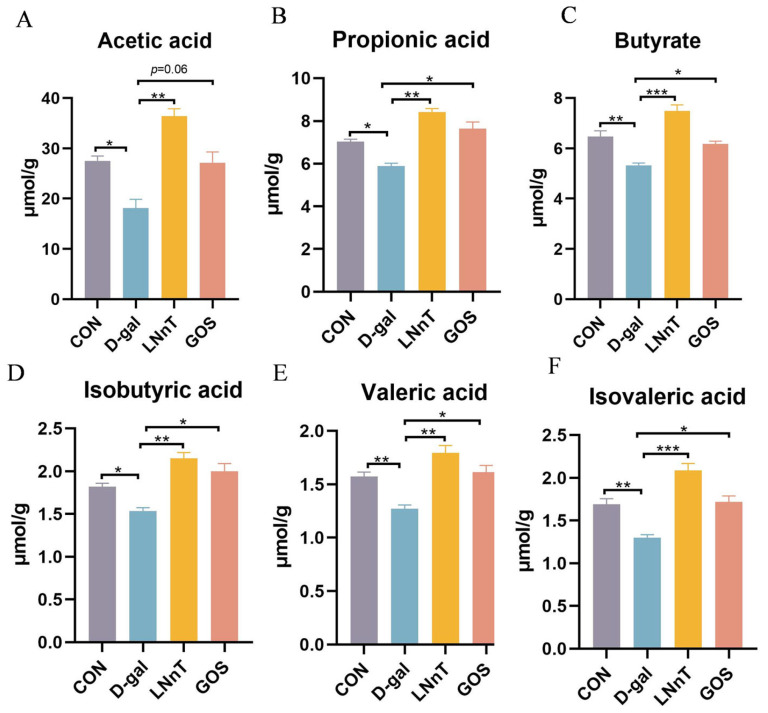
Effect of LNnT on SCFAs levels in mouse feces. (**A**) Acetic acid. (**B**) Propionic acid. (**C**) Butyrate. (**D**) Isobutyric acid. (**E**) Valeric acid. (**F**) Isovaleric acid. All data were presented as means ± SEM (*n* = 5). * *p* < 0.05, ** *p* < 0.01, *** *p* < 0.001 vs. D-gal group using one-way ANOVA followed by Duncan’s multiple range test.

Concentrations of key SCFAs, including acetic acid, propionic acid, butyric acid, isobutyric acid, valeric acid, and isovaleric acid, were quantified in fecal samples from each group. As shown in Figure 5A–F, the D-gal group exhibited a significant reduction in all detected SCFAs compared to the control group (*p* < 0.05). In contrast, both LNnT and GOS interventions effectively restored SCFA levels, resulting in significant increases relative to the D-gal group (*p* < 0.05). SCFAs, particularly butyrate, have been extensively documented to enhance intestinal barrier function by upregulating tight junction proteins (e.g., claudin-1, occludin, ZO-1) and exerting anti-inflammatory effects [43,44,45], suggesting that the observed increase in SCFAs may contribute to the improved colonic barrier integrity and reduced inflammation in LNnT-treated mice.

### 2.6. LNnT Alleviates D-Gal-Induced Sphingolipid Metabolic Disorder

**Figure 6:**
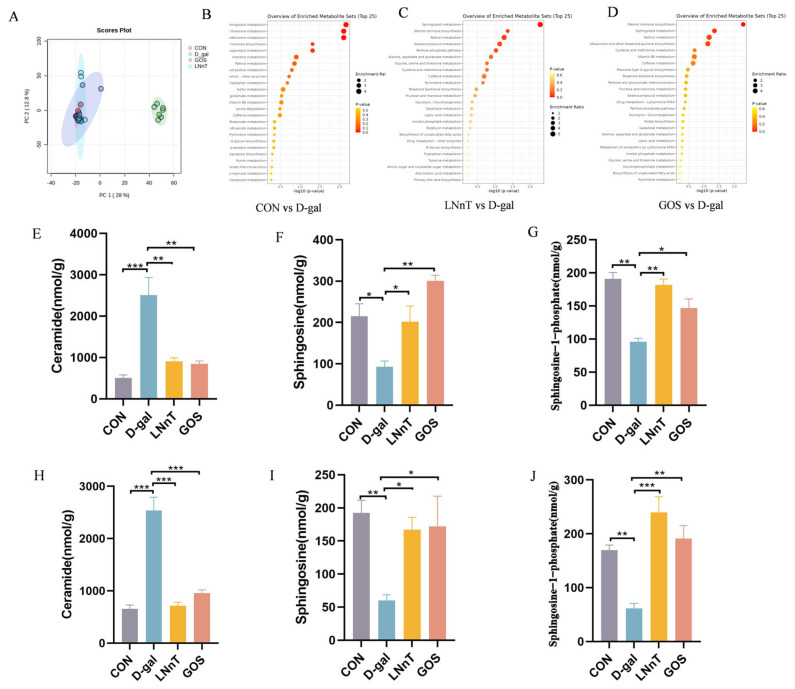
Effect of LNnT on feces metabolites in mice. (**A**) PCA diagram of each group. (**B**) The metabolic pathways of feces differential metabolites between control and D-gal group. (**C**) The metabolic pathways of feces differential metabolites between LNnT and D-gal group. (**D**) The metabolic pathways of feces differential metabolites between GOS and D-gal group. Changes in the Levels of Ceramide (**E**), Sphingosine (**F**), and Sphingosine-1-Phosphate (**G**) in Mouse colon tissue. Changes in the Levels of Ceramide (**H**), Sphingosine (**I**), and Sphingosine-1-Phosphate (**J**) in Mouse feces. All data were presented as means ± SEM (*n* = 5). * *p* < 0.05, ** *p* < 0.01, *** *p* < 0.001 vs. D-gal group using one-way ANOVA followed by Duncan’s multiple range test.

To investigate the underlying mechanism of LNnT in D-gal-induced mice, untargeted metabolomics analysis was performed on fecal samples. PCoA revealed distinct separation between the control and D-gal groups, indicating significant D-gal-induced perturbations in host metabolic profiles. Notably, both LNnT and GOS intervention groups clustered separately from the D-gal group and overlapped more closely with the control group, suggesting that LNnT and GOS mitigate metabolic disturbances and promote the restoration of physiological homeostasis (Figure 6A). To further validate the metabolic discrimination between groups, OPLS-DA was applied. As shown in Figure S4, the OPLS-DA models for control vs. D-gal, LNnT vs. D-gal, and GOS vs. D-gal all exhibited excellent performance, with R^2^Y/Q^2^ values of 0.997/0.974, 0.994/0.954, and 0.988/0.941, respectively, confirming the validity of all models without overfitting. Subsequently, differential metabolites were screened between the control vs. D-gal groups and LNnT vs. D-gal groups using the criteria of *p* < 0.05 and variable importance in projection (VIP) > 1, followed by Kyoto Encyclopedia of Genes and Genomes (KEGG) pathway enrichment analysis. Compared with the control group, the D-gal group exhibited significant dysregulation in sphingolipid metabolism (*p* = 0.006), glycine/serine/threonine metabolism (*p* = 0.007), cysteine/methionine metabolism (*p* = 0.007), and steroid hormone biosynthesis (*p* = 0.04) (Figure 6B). These findings suggest that D-gal may induce intestinal injury and inflammation by disrupting amino acid homeostasis and lipid-mediated signaling pathways. In the LNnT vs. D-gal comparison, sphingolipid metabolism (*p* = 0.005) and steroid hormone biosynthesis (*p* = 0.04) were the most significantly enriched pathways (Figure 6C).

To confirm these observations, we quantitatively assessed key sphingolipid metabolites in colon tissues (Figure 6E–G) and fecal samples (Figure 6H–J). Relative to the control group, the D-gal group showed significantly elevated ceramide levels *(p* < 0.05) and reduced concentrations of sphingosine and its phosphorylated derivative S1P (*p* < 0.05). Both LNnT and GOS administration effectively counteracted these changes, restoring metabolite levels to those observed in the control group (*p* < 0.01). Collectively, these findings indicate that LNnT can rectify the sphingolipid metabolic imbalance induced by D-gal.

### 2.7. LNnT Improves Colonic Barrier Function via the ACER2/SphK2/S1P Axis

**Figure 7:**
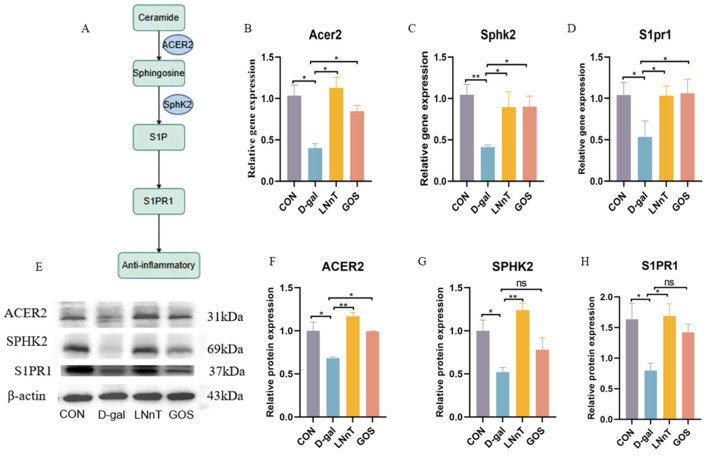
Effect of LNnT on the expression of key molecules in the sphingolipid metabolism pathway in colon tissue of D-galactose-induced mice. (**A**) Schematic diagram of the sphingolipid mechanism. Relative mRNA expression levels of ACER2 (**B**), SphK2 (**C**), and S1PR1 (**D**) in colon tissue. (**E**) Representative Western blot bands of ACER2, SphK2, S1PR1, and β-actin. Quantitative analysis of relative ACER2 (**F**), SphK2 (**G**), and S1PR1 (**H**) protein expression. All data were presented as means ± SEM (*n* = 5). * *p* < 0.05, ** *p* < 0.01, vs. D-gal group using one-way ANOVA followed by Duncan’s multiple range test. ns, not significant.

To further elucidate the molecular mechanism underlying LNnT-mediated regulation of sphingolipid metabolism, we analyzed the expression of key molecules in this pathway using qPCR and Western blot (WB), with a schematic overview provided in Figure 7A. The results showed that D-gal significantly downregulated both the mRNA and protein levels of alkaline ceramidase 2 (ACER2, catalyzes ceramide hydrolysis to sphingosine) and sphingosine kinase 2 (SphK2, phosphorylates sphingosine to S1P) compared to the control group (Figure 7B–H, *p < 0.05*). LNnT intervention effectively reversed these changes, restoring ACER2 and SphK2 protein expression to near-normal levels (*p* < 0.05). Consistent with the protein data, LNnT also significantly increased the mRNA expression of ACER2 and SphK2 in colon tissue (*p* < 0.05). Furthermore, LNnT upregulated the expression of the S1P receptor 1 (S1PR1) at both the mRNA and protein levels.

Notably, while GOS intervention significantly altered the mRNA expression of ACER2 and SphK2, its effects on SphK2 and S1PR1 protein expression did not reach statistical significance. These findings suggest that LNnT alleviates D-gal-induced colonic barrier dysfunction primarily by modulating the ACER2/SphK2/S1P/S1PR1 signaling axis, thereby reestablishing sphingolipid metabolic homeostasis.

### 2.8. Correlation Analysis Between Key Regulators of Intestinal Barrier Function and Gut Microbiota in D-Gal-Induced Mice

**Figure 8:**
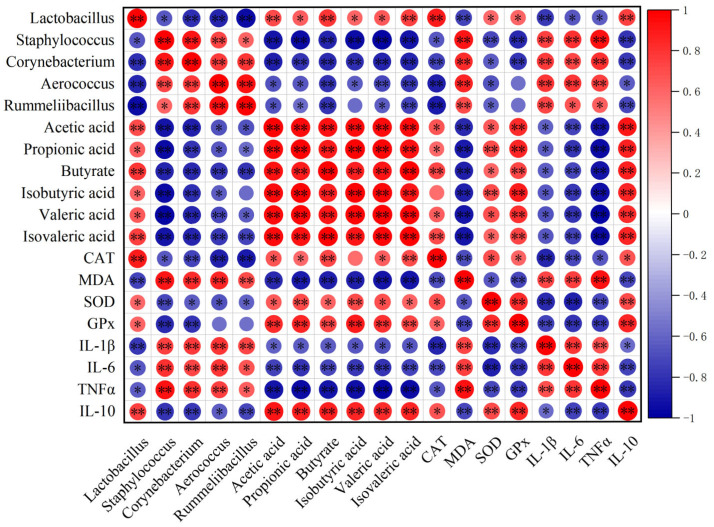
Spearman correlation analysis between key regulatory factors in the regulation of intestinal barrier function and gut microbiota at the family level in D-galactose-induced aging mice. * *p* < 0.05, ** *p* < 0.01.

To explore potential associations between gut microbiota and host physiological parameters, Spearman correlation analysis was performed. As shown in Figure 8, the D-gal group exhibited a significant increase in the abundance of *Staphylococcaceae* and *Corynebacteriaceae*. These microbial alterations were positively correlated with pro-inflammatory cytokines (TNF-α, IL-6, IL-1β) and malondialdehyde (MDA), and negatively correlated with the anti-inflammatory cytokine IL-10, antioxidant enzymes (SOD, CAT, GPx), and various SCFAs. These associations suggest a potential link between gut microbial composition and systemic inflammation and oxidative stress in D-gal-induced mice. Notably, LNnT and GOS interventions reversed these correlation patterns.

## 3. Discussion

This study investigated the protective effects and underlying mechanisms of the human milk oligosaccharide LNnT against D-gal-induced intestinal dysfunction and cognitive impairment in mice. Results demonstrated that LNnT intervention effectively mitigated D-gal-induced systemic physiological abnormalities (including cognitive decline), structural damage to the colonic barrier, systemic inflammation and oxidative stress, gut microbiota dysbiosis, and disturbances in sphingolipid metabolism. The protective effects of LNnT were multi-faceted and systemic. Although GOS intervention also demonstrated certain beneficial effects, LNnT showed superior efficacy in regulating gut microbiota composition, balancing sphingolipid metabolic pathways, and restoring intestinal barrier function.

The D-gal-induced model serves as a widely used tool for studying chronic injury and associated functional impairments, simulating pathophysiological processes by promoting oxidative stress and metabolic disorders [46,47]. Experimental results indicate that D-gal leads to impaired weight gain in mice and pathologically elevated indices of key organs (liver, kidney, spleen)—findings consistent with the systemic stress state induced by this model. Notably, both LNnT and GOS interventions effectively alleviated these abnormal phenotypes, preliminarily confirming their potential to improve systemic pathological states. Regarding body weight dynamics, although LNnT and GOS interventions delayed the onset of D-gal-induced weight loss and significantly mitigated its severity, they did not completely prevent the decline. This partial effect aligns with the complex pathophysiology of D-gal-induced subacute injury, which involves systemic oxidative stress, mitochondrial dysfunction, and metabolic disturbances that are difficult to fully reverse with a single dietary intervention [14]. Notably, the body weights of the LNnT and GOS groups remained significantly higher than those of the D-gal group throughout the experiment and were closer to control levels, indicating that LNnT exerts a meaningful, albeit partial, protective effect by alleviating D-gal-induced tissue damage and metabolic disorders. These findings preliminarily confirm the potential of LNnT to improve systemic pathological states.

Cognitive behavioral assessments provided crucial functional evidence for the systemic protective effects of LNnT. Through Y-maze and MWM tests, this study revealed that D-gal model mice exhibited significant impairments in spatial learning and memory. Specifically, in the Y-maze test, D-gal treatment significantly reduced total movement distance (5.82% decrease, *p* < 0.001), novel arm exploration time (11.21% decrease, *p* < 0.01), and novel arm exploration distance (10.25% decrease, *p* < 0.05) compared to the control group. In the MWM test, D-gal-treated mice showed prolonged escape latency (approximately 2.3-fold increase on day 5, *p* < 0.05), fewer platform crossings (60.0% decrease, *p* < 0.01), and reduced time spent in the target quadrant (10.16% decrease, *p* < 0.05). These behavioral deficits are consistent with the features of D-gal-induced neurological dysfunction models [48,49]. Importantly, LNnT intervention significantly ameliorated all these behavioral deficits (*p* < 0.05), effectively reversing cognitive decline. Concurrently, LNnT intervention restored intestinal barrier integrity, alleviated systemic inflammation and oxidative stress, and reshaped a favorable microbiota–metabolite profile, including the restoration of SCFAs levels and the rebalancing of sphingolipid metabolism. Collectively, these improvements created a more supportive microenvironment for the brain, suggesting an association between the amelioration of intestinal and systemic pathology and the protection of higher-order brain functions, with the gut–brain axis as a potential underlying pathway, although causality remains to be established.

Disruption of intestinal barrier integrity is a common feature and driving factor in multiple chronic pathological states [4]. Studies have shown that D-gal induces inflammatory infiltration, mucosal structural damage, and shallower crypt depth in mouse colons, while significantly downregulating the expression of TJ proteins (Claudin-1, Occludin, ZO-1). TJ proteins form the molecular basis for maintaining selective permeability in the intestinal epithelium, and their downregulation serves as a key indicator of intestinal barrier leakage [50]. Both LNnT and GOS significantly reversed these pathological changes, demonstrating their efficacy in enhancing the integrity of the intestinal mechanical barrier. This finding aligns with the emerging research trend indicating that certain human milk oligosaccharides possess barrier-protective functions [51,52].

Chronic inflammation and oxidative stress represent core pathological mechanisms driving tissue damage [53]. In this study, D-gal group mice exhibited elevated proinflammatory factors (IL-1β, IL-6, TNF-α), reduced anti-inflammatory factor IL-10, decreased antioxidant enzyme activity (SOD, CAT, GPx), and accumulation of oxidative damage marker MDA. LNnT intervention reversed this imbalance, demonstrating anti-inflammatory and antioxidant effects. It is noteworthy that LNnT demonstrated superior efficacy to GOS in significantly elevating IL-10 levels, suggesting a potential unique advantage in fine-tuning immunoregulatory balance.

The gut microbiota and its metabolites play a pivotal role in maintaining intestinal homeostasis. Sequencing results revealed that D-gal caused a decrease in α- and β-diversity of the gut microbiota, along with structural disruption, manifested as a reduction in beneficial bacteria (such as Lactobacillus) and an increase in potentially harmful bacteria (such as Staphylococcus). This dysbiosis pattern resembles the microbiome characteristics observed in many chronic disease states [53]. LNnT intervention effectively restored microbial diversity and beneficial bacterial abundance. LNnT significantly elevated levels of SCFAs in feces, including acetate, propionate, and butyrate. SCFAs are known to serve as an energy source for colonic epithelium and have been reported to exert anti-inflammatory and barrier-maintaining effects through multiple mechanisms [54]. LEfSe analysis identified that LNnT intervention enriched specific taxa, including Betaproteobacteria and Sutterella. While these genera have been previously associated with various pathological conditions [55,56], emerging evidence suggests a more nuanced, context-dependent role for these bacteria. Recent studies have demonstrated that Sutterella exhibits a “double-edged sword” effect in the gut–brain axis, possessing both pro-inflammatory properties (e.g., inducing IL-6 and TNF-α) and immunomodulatory potential (e.g., promoting anti-inflammatory IL-10 production) depending on the host microenvironment [57]. Additionally, Betaproteobacteria have been identified as marked taxa regulated by metabolite sensing (e.g., GPR35-mediated kynurenic acid signaling) that contribute to maintaining gut homeostasis in inflammatory conditions [58]. The enrichment of these taxa in the LNnT-treated group, alongside the significant elevation of systemic IL-10 and reduction in pro-inflammatory cytokines, suggests that their expansion may occur within a broader context of immune balance restoration rather than simply reflecting pathogenic dysbiosis. Thus, by modulating the gut microbiota through prebiotic-like actions and promoting the production of beneficial metabolites such as SCFAs, LNnT contributes importantly to the protection of intestinal barrier function. Notably, these findings establish a strong correlation between LNnT-induced gut microbiota remodeling and the restoration of sphingolipid metabolic homeostasis. Emerging evidence from fecal microbiota transplantation (FMT) studies has demonstrated that the gut microbiota plays a causal role in regulating host sphingolipid metabolism [59,60,61]. For instance, successful FMT in patients with recurrent Clostridioides difficile infection induces significant shifts in the sphingolipid lipidome, characterized by elevated trihydroxy ceramides post-FMT [59]. Conversely, disruption of gut microbiota by antibiotic treatment leads to sphingolipid accumulation and altered lipid absorption in aged mice [62]. Furthermore, specific bacterial taxa, including Bacteroides and Bifidobacterium species, are capable of de novo sphingolipid synthesis, thereby influencing host metabolic and immune homeostasis [63]. A recent study using FMT from depressed patients into germ-free mice provided direct evidence that gut microbiota can induce depressive phenotypes by modulating central glycerophospholipid and sphingolipid metabolism via the gut–brain axis [64]. Collectively, these findings support the existence of a “gut microbiota–sphingolipid metabolism axis” and suggest its potential as a therapeutic target. However, the present study is observational in nature; while the correlational data strongly suggest, and the FMT literature supports, a causal link between LNnT-mediated gut microbiota modulation and improved sphingolipid metabolism, direct experimental validation is warranted. Future investigations employing fecal microbiota transplantation from LNnT-treated donors into germ-free or antibiotic-pretreated recipient mice will be essential to definitively establish causality.

Untargeted metabolomics directed our focus to the sphingolipid metabolic pathway. Results revealed that D-gal led to ceramide accumulation in colonic tissue and feces, while sphingosine and its active metabolite S1P decreased. The dynamic balance between ceramide and S1P—conceptualized as the “sphingolipid rheostat”—is critical for cell survival, inflammation, and barrier function [65]. Excessive ceramide promotes oxidative stress, inflammation, and apoptosis, whereas S1P predominantly transmits signals that support cell survival, enhance barrier integrity, and exert anti-inflammatory effects [66]. Mechanistic investigations revealed that D-gal downregulated the expression of ACER2, a key enzyme in ceramide degradation, and SphK2, a key enzyme in S1P synthesis. LNnT intervention specifically upregulates the gene and protein expression of ACER2 and SphK2, thereby restoring the ceramide-S1P metabolic axis, accompanied by increased expression of the S1P receptor S1PR1. By binding to S1PR1, S1P stabilizes the cytoskeleton and promotes tight junction assembly, thereby directly enhancing epithelial barrier function [67,68]. Based on the observed expression changes, this study proposes a potential axis through which LNnT may exert its effects: upregulation of ACER2/SphK2, restoration of S1P production and signaling, and enhancement of tight junctions and barrier function. However, causality remains to be established through functional validation. Although GOS showed some regulatory effects at the gene transcription level, its impact on the expression of key proteins (SphK2, S1PR1) was not significant, which may partly explain its inferior restorative efficacy compared to LNnT.

Correlation analysis further integrated the above findings: increased harmful bacteria in the D-gal group correlated positively with pro-inflammatory factors and oxidative damage, while negatively correlating with beneficial metabolites SCFAs; LNnT intervention reversed these detrimental associations. This strongly supports the notion that LNnT exerts its protective effects by remodeling a healthy “microbiota–metabolite–host” interaction network. An intriguing observation is that Bacteroides, which was enriched by LNnT, is a known commensal bacterium capable of synthesizing sphingolipids in the gut [69], providing a clue to the hypothesis that LNnT may indirectly influence host sphingolipid metabolism by modulating specific microbial taxa.

## 4. Materials and Methods

### 4.1. Materials

LNnT, with a purity of >92%, was provided by Inner Mongolia Yili Industrial Group Co., Ltd. (Hohhot, China). The remaining ~8% of the preparation consists primarily of structurally related human milk oligosaccharides, including lacto-N-triose II (LNT2) and lacto-N-tetraose (LNT), which are naturally occurring biosynthetic intermediates or structural analogs of LNnT. GOS (purity > 98%) was purchased from Shanghai Maclin Biochemical Technology Co., Ltd. (Shanghai, China), with a purity greater than 99%. D-galactose was purchased from Beijing Solarbio Science & Technology Co., Ltd. (Beijing, China). IL-1β, TNF-α, IL-6, IL-10, CAT, SOD, MDA, and GPX kits were purchased from Shenzhen Xinbosheng Biotechnology Co., Ltd. The antibodies ACER2, SphK2, and S1PR1 were purchased from Nanjing Baoduo Biotechnology Co., Ltd. (Nanjing, China), and Abbo Antigen Shanghai Trading Co., Ltd. (Shanghai, China).

### 4.2. Animals

Eight-week-old male ICR mice (38.0 ± 1.0 g) were obtained from SPF (Beijing, China). The animals were housed in a controlled environment with a temperature of 22 °C, relative humidity of 55% ± 5%, and a 12 h light/dark cycle. After a one-week acclimatization period, the 32 mice were individually numbered, and a randomization sequence was generated using the random number table method, then randomly assigned to four groups: the control group, the D-gal group, the LNnT group, and the GOS group, with eight mice in each group. Starting from day 8, the three experimental groups received daily intraperitoneal injections of D-galactose (150 mg/kg body weight) for six consecutive weeks to establish the model, based on previously described protocols with modifications [17,70]. The control group was intraperitoneally injected with the same volume of normal saline. During the same period, the LNnT and GOS groups were given 300 mg/kg of LNnT and GOS by oral gavage, while the control and D-gal groups received the same volume of saline by the same method. All animals had free access to food and water and their body weight was recorded every 7 days. All animal experiments were conducted in accordance with the Chinese Guidelines for the Ethical Review of Laboratory Animal Welfare (GB/T 35892-2018) and were approved by the Ethics Committee of Nanchang University (License number: 0064257).

### 4.3. Behavior Test

#### 4.3.1. Y-Maze Test

The Y-maze test assesses the spatial memory ability of rodents by their natural tendency to explore novel environments [71]. The Y-maze consists of three identical stainless steel arms (50 cm × 20 cm × 10 cm) arranged at a 120° angle to one another, named arm A, B, and C. During the training phase, arm A (the novel arm) was blocked with a barrier, allowing the animal free access to explore only arms B and C. Each mouse was placed at arm B and permitted to explore the available arms for 10 min before being removed from the maze. After an interval of 2 h, all arms were made fully accessible, and the mouse was allowed to freely explore for 5 min. Parameters analyzed during the test phase included total distance moved, movement time, and distance traveled within the novel arm, as well as the spontaneous alternation rate, calculated as the percentage of triads of consecutive arm entries in which all three arms were different.

#### 4.3.2. Water Maze Test

The MWM test was employed to evaluate spatial learning and memory in the rodents. The experimental procedure was based on previous research [72], and the specific procedures are as follows: The water maze device (manufactured by NJKEWBIO, Nanjing, China) had a diameter of 1.5 m and a height of 35 cm. Distinctly colored and shaped visual markers were affixed to the pool walls, which was divided into four quadrants. On the day before the formal experiment (day 0), a platform was placed in one quadrant, raised approximately 2 cm above the water surface, and each mouse was acclimatized to each quadrant for 1 min. Over the 5 days of learning trials (days 1–5), the water in the pool was tinted with non-toxic black dye and maintained at 23 ± 1 °C, and the platform was submerged about 1 cm below the water surface, maintaining a fixed position. During the training trials, each mouse was placed alone on a small platform within the target quadrant for 20 s, and they were trained to find the target platform within 1 min. If a mouse failed to find the platform within the specified time, it would be guided to the target platform and allowed to stay there for 20 s. Each mouse underwent four trials per day, starting from four different positions around the water tank each time, with a 25-min interval between each test.

On day 6, the hidden platform was removed, and each mouse was allowed to search for the platform during the probe trial. Throughout the learning trials, the time required to reach the target platform (escape latency) and the total distance traveled were recorded using video tracking and behavioral analysis software (KEMaze, NJKEBIO, Nanjing, China). In addition, during the probe trial, the percentage of time spent in the target quadrant and the percentage of distance traveled within that quadrant were measured.

### 4.4. Experimental Design and Sample Collection

After the completion of the behavioral experiments, mice were anesthetized using isoflurane to minimize suffering [73]. Blood samples were collected via orbital bleeding and centrifuged to isolate serum. After euthanasia, colonic contents were immediately collected and stored at −80 °C for subsequent 16S rRNA sequencing to analyze gut microbiota composition. The spleen, kidney, and liver were excised and weighed. Remaining tissue samples were rapidly frozen on dry ice and transferred to a −80 °C ultra-low temperature freezer for future molecular biological or biochemical analyses.

### 4.5. Morphological Studies

Freshly harvested colon tissues were fixed in 4% paraformaldehyde before paraffin embedding. Subsequently, tissue sections were dewaxed and rehydrated, followed by staining with hematoxylin-eosin (H&E). After staining, the slides were dehydrated and protected with neutral resin. All sections were evaluated using an optical microscope (Olympus IX53, Tokyo, Japan) in a blinded manner and further analyzed with ImageJ software (version 1.54, Media Cybernetics, Rockville, MD, USA).

### 4.6. Immunohistochemistry

Colon tissues fixed in 4% paraformaldehyde were embedded in paraffin and sectioned at a thickness of approximately 5 μm. Antigen retrieval was performed using citrate sodium buffer (pH 6.0) under microwave heating. Subsequently, nonspecific binding was blocked with 5% goat serum for 30 min. The sections were incubated with primary antibodies (anti-Occludin, anti-Claudin-1, anti-ZO-1) overnight at 4 °C. After washing three times with PBS, secondary antibody was applied and incubated at 37 °C for 1 h. Staining was developed using 3,3′-diaminobenzidine (DAB), and hematoxylin counterstaining was performed prior to microscopic examination and image acquisition.

### 4.7. Analysis of Short-Chain Fatty Acids

An HP-FFAP chromatographic column (30 m × 0.32 mm × 0.25 μm, Agilent Technologies, Santa Clara, CA, USA) was used to analyze short-chain fatty acids (SCFAs) in fecal samples. Approximately 10 mg of fecal sample was weighed and homogenized in PBS buffer (0.01 M, pH 7.2–7.4) using a homogenizer, followed by sonication for 10 min (100 W). Subsequently, 20 μL of 10% sulfuric acid was added, and vortexed for 30 s, after which 800 μL of diethyl ether was added and vortexed again for 30 s to extract fatty acids. The mixture stood for 20 min and then underwent centrifugation for 20 min (10,000× *g*, 4 °C). The upper ether phase was collected into a new centrifuge tube and nitrogen blowing was performed to concentrate it to 500 μL. A 0.22 μm organic phase filter membrane was used for filtration and the mixture was transferred to a brown injection vial. SCFA content was analyzed by gas chromatography–mass spectrometry (GC–MS). It was automatically injected into the injection port at a split ratio of 20:1 (1 μL) at 270 °C. The column temperature was programmed as follows: stabilize at 140 °C for 3 min, then increase to 190 °C at a rate of 6 °C/min, and maintain for 5 min. Quantification of SCFAs levels in the fecal samples was performed using an external standard calibration curve.

### 4.8. Intestinal Microbiota Analysis

Colonic content samples (*n* = 5) were subjected to 16S rDNA gene sequencing analysis provided by Personal Biotechnology Co., Ltd. (Shanghai, China), according to the manufacturer’s guidelines, using the EZNA Bacterial DNA Kit (OMEGA, New York, NY, USA) to extract microbial genomic DNA from these samples. Subsequently, each DNA sample (30 ng) was combined with corresponding fusion primers in a PCR reaction system to amplify the V3–V4 region of the rDNA. Amplification products were purified using Vazyme VAHTSTM DNA Clean Beads and dissolved in elution buffer for library construction. Following library preparation, community DNA fragments were sequenced on the Illumina MiSeq/NovaSeq platform using a paired-end approach. Raw fastq files were quality-filtered and merged using Quantitative Insights into Microbial Ecology 2 (QIIME2, version 2022.11). Sequences were clustered into operational taxonomic units (OTUs) at a 97% similarity threshold using VSEARCH (v2.13.4) to reduce data complexity and mitigate the impact of sequencing errors. Subsequently, representative sequences from each OTU were subjected to taxonomic classification using the classify-sklearn naive Bayes classifier in the Feature Classifier plugin, with reference to the Silva v132 99% OTU database. This two-step approach—first clustering into OTUs, then classifying representative sequences against a reference database—was adopted to balance computational efficiency with taxonomic resolution. The subsequent analyses include species composition analysis, assessment of A and B diversity, as well as species difference analysis and marker species analysis based on OTUs and species annotation results, and the use of linear discriminant analysis (LDA) effect size (LEfSe) analysis to determine significant differences between groups (LDA > 3.0).

### 4.9. Reverse Transcription-Quantitative PCR

To investigate the expression of colon genes associated with specific pathways, a set of target genes was selected for analysis (Table S1). Total RNA was extracted from colon tissues using Trizol reagent (Invitrogen, Carlsbad, CA, USA). Following extraction, 1 μg of RNA was reverse-transcribed into cDNA using a Takara kit (Takara, Shiga, Japan). The reverse transcription reaction system and the subsequent PCR cycling conditions were performed with reference to established methodological protocols [29]. The cDNA was then amplified and detected using a real-time quantitative PCR system (Bio-Rad, Hercules, CA, USA). GAPDH was used as the internal reference, and the relative mRNA expression levels of each gene were calculated using the 2^−ΔΔCt^ method.

### 4.10. Untargeted Metabolomics Analysis

A 100 mg sample was weighed and mixed with pre-cooled 80% methanol aqueous solution (*v*/*v*) at a ratio of 1 mL per 100 mg of tissue. The mixture was placed in a grinding tube with steel beads and homogenized using a homogenizer through two cycles of grinding. Following homogenization, the sample was kept at −20 °C for 1 h, then centrifuged for 10 min (13,000 r, 4 °C). The supernatant was collected and recentrifuged under identical conditions for 5 min to enhance purity. Finally, the supernatant was filtered through a 0.22 μm organic phase membrane and transferred into a brown injection vial. The primary metabolites profiled in the feces were identified by the Q Exactive Focus system equipped with a Thermo-Accucore C18 column (2.1 × 100 mm; 2.6 μm, Waters, Waltham, MA, USA). The mobile phase consisted of water containing 0.1% formic acid (A) and acetonitrile containing 0.1% formic acid (B). The elution program was set to a linear increase of phase B from an initial 2% to a specified final concentration over 15 min, with a flow rate of 0.4 mL/min, a column temperature of 40 °C, and an injection volume of 2 μL. Parameters for the electrospray ionization (ESI) source were configured as follows: spray voltage of 2.75 kV, capillary temperature of 325 °C, and auxiliary gas heater temperature of 400 °C; full scan mass range 80–1000 m/z at a resolution of 70,000; sheath gas flow rate was 60 mL/min, auxiliary gas flow rate was 10 mL/min, and sweep gas flow rate was 1 mL/min. Raw data were processed using Progenesis QI (v 2.4) for peak extraction, alignment, and quantification. Metabolites were initially annotated against the Human Metabolome Database (HMDB). Multivariate statistical analysis was performed using Metaboanalyst, including principal component analysis (PCA) and orthogonal partial least squares-discriminant analysis (OPLS-DA). To evaluate the robustness of the OPLS-DA model and avoid overfitting, model quality was assessed using cumulative R^2^Y (goodness of fit) and Q^2^ (goodness of prediction) values. R^2^Y and Q^2^ values close to 1 indicate strong explanatory power and predictive ability. Variable importance in projection (VIP) values was calculated to evaluate the contribution of metabolites to group discrimination. Differentially abundant metabolites were screened using the criteria: VIP > 1, *p* < 0.05 (Student’s *t*-test), and absolute fold change > 2. Significantly altered metabolites were subsequently subjected to KEGG pathway annotation and enrichment analysis.

### 4.11. Quantitative Analysis of Ceramides, Sphingosine, and Sphingosine-1-Phosphate (S1P)

Analysis was carried out on an HPLC-ESI-MS/MS system equipped with a ZORBAX Eclipse XDB-C18 column (2.1 × 100 mm, 3.5 μm). Samples were dissolved in a chloroform/methanol mixture (1:1, *v*/*v*), filtered through a 0.22 μm organic membrane, and 2 μL of the filtrate was injected into the system. The mobile phase consisted of 0.04% formic acid in water (solvent A) and 0.02% formic acid in acetonitrile (solvent B), delivered at a flow rate of 0.6 mL/min. The following gradient elution program was applied: 5% B (0–0.4 min), increased linearly to 95% B (0.4–4 min), maintained at 95% B (4–12 min), returned linearly to 5% B (12–20 min). Analytes were identified by matching retention times with certified standards. Quantification of ceramide, sphingosine, and S1P in fecal samples was performed using external calibration curves.

### 4.12. Western Blot

The colon tissues were collected, 1 mL of lysis buffer (containing 900 μL RIPA and 10 μL phenylmethylsulphonyl fluoride) was added, then tissues were lysed on ice for 30 min, followed by homogenization. The homogenate was centrifuged at 12,000 rpm and 4 °C for 15 min. After collecting the supernatant, the protein concentration was determined using a BCA kit (Beyotime Biotechnology Co., Ltd., Shanghai, China). Subsequently, 6 × Loading buffer (6 × concentrate; Beyotime, Shanghai, China) was added to sample, which was then denatured at 95 °C for 5 min and stored at −20 °C. Equal amounts of protein samples were subjected to electrophoresis using 10% sodium dodecyl sulfate-polyacrylamide gel electrophoresis (SDS-PAGE). Next, the proteins were transferred to polyvinylidene difluoride (PVDF) membranes. The membrane was blocked with 5% nonfat milk for 2 h and washed three times with Tris-buffered saline containing Tween 20 (TBST). After that, the membrane was incubated at 4 °C for 16 h, with the following primary antibodies: anti-ACER2 (Nanjing Baoduo Biotechnology Co., Ltd., Nanjing, China, Cat# BZ16068r, 1:750), anti-SphK2 (Abbo Antigen Shanghai Trading Co., Ltd., Shanghai, China, Cat# ab320741, 1:1000), anti-S1PR1 (Abbo Antigen Shanghai Trading Co., Ltd., Cat# ab233386, 1:1000), and anti-β-actin (Beyotime Biotechnology, Cat# S10927, 1:3000). After primary antibody incubation, the membrane was washed three times with TBST (10 min each). Subsequently, the membrane was incubated with an appropriate horseradish peroxidase (HRP)-conjugated secondary antibody (goat anti-rabbit or goat anti-mouse, 1:3000 dilution) for 2 h at room temperature. The blots were visualized using an enhanced chemiluminescence (ECL, Millipore, Billerica, MA, USA) substrate, and the integrated optical density (IOD) values were measured using Image J software. The levels of β-actin and GAPDH were used as internal reference proteins.

### 4.13. Statistical Analysis

Statistical analysis was performed using IBM SPSS software version 19. The results of the single factor analysis of variance (ANOVA) were expressed as mean ± standard error of the mean (SEM). The Duncan multiple range test was utilized for group comparisons. *p* < 0.05 was considered statistically significant.

## 5. Limitations

Although this study has yielded a series of meaningful findings, several limitations should be acknowledged and addressed in future investigations. First, due to experimental constraints, the sample sizes employed in this study were relatively limited. Such sample scales may constrain statistical power to a certain extent, particularly for analyses such as gut microbiota profiling and metabolomics, which are inherently subject to high biological variability. Therefore, the robustness of the findings would benefit from further validation in studies with larger cohorts. Second, the study was conducted exclusively using male ICR mice, with no inclusion of female subjects. Given that sex is a well-recognized biological variable influencing gut physiology, microbiota composition, and cognitive function, the current findings derived solely from male animals may not be directly generalizable to female populations, nor do they allow for assessment of potential sex-dependent differences in the protective effects of LNnT. Third, the current mechanistic exploration largely relies on correlative evidence and lacks direct causal validation. For instance, the interplay between gut microbiota and sphingolipid metabolism, as well as the specific regulatory role of the ACER2/SphK2/S1P/S1PR1 signaling axis, have yet to be substantiated through functional experiments such as fecal microbiota transplantation, gene knockdown, or pharmacological inhibition. Consequently, the related conclusions require further support from functional validation studies. Moreover, the hypothesis that LNnT improves cognitive function via the gut–brain axis currently lacks direct neurological evidence, such as assessments of neuroinflammatory markers in brain tissue, hippocampal histopathological features, or brain-derived neurotrophic factor levels. Finally, the D-galactose-induced model employed in this study is essentially an accelerated aging model driven by oxidative stress and the accumulation of advanced glycation end products. This model may not fully recapitulate the pathophysiological processes underlying natural aging or human diseases characterized by intestinal barrier dysfunction and gut–brain axis dysregulation. Therefore, the translational relevance of the findings warrants cautious interpretation and further validation using more physiologically relevant models.

## 6. Conclusions

This study demonstrates that LNnT exerts protective effects against D-galactose-induced intestinal barrier dysfunction and associated cognitive impairment in mice. These protective effects are multifaceted: LNnT restored colonic barrier integrity by upregulating tight junction proteins (Claudin-1, Occludin, ZO-1), alleviated systemic inflammation and oxidative stress, and improved spatial learning and memory performance in both the Y-maze and Morris water maze tests.

Further analysis revealed that these beneficial effects were associated with remodeling of the gut microbiota, characterized by increased microbial diversity, enrichment of beneficial bacterial taxa, and elevated fecal short-chain fatty acid levels. Untargeted metabolomics further identified sphingolipid metabolism as a key pathway, with LNnT reducing colonic ceramide accumulation, restoring sphingosine and S1P levels, and upregulating the ACER2/SphK2/S1PR1 signaling axis.

Despite the significance of these findings, several limitations should be acknowledged, including the relatively small sample size, exclusive use of male mice, lack of direct mechanistic validation (e.g., enzyme inhibitors, fecal microbiota transplantation), and the correlational nature of the microbiota and metabolomics data. Future studies incorporating larger cohorts, female subjects, and targeted functional interventions are warranted to establish causality and facilitate the translation of these findings toward potential nutritional applications.

## Data Availability

Data are contained within the article and Supplementary Materials.

## Supplementary Materials

The following supporting information can be downloaded at: https://www.mdpi.com/article/10.3390/molecules31091410/s1, Figure S1: Spontaneous alternation in the Y-maze (A), total swimming distance (B), and average swimming speed (C) in the Morris water maze; Figure S2: Venn diagram; Figure S3: NMDS diagram; Figure S4: OPLS-DA score plots of metabolic profiles. (A,B) Control vs. D-gal group; Table S1: Primer Information for qPCR.
