# Supplementary material for: Human Milk Oligosaccharide LNnT Attenuates Colonic Barrier Dysfunction and Associated Cognitive Impairment via Modulating Sphingolipid Metabolism and Gut Microbiota
Source: Molecules. 2026 Apr 24;31(9):1410. doi: 10.3390/molecules31091410 (PMC13165385; doi:10.3390/molecules31091410)

Supporting Information

Human Milk Oligosaccharide LNnT Attenuates Colonic Barrier Dysfunction and Associated Cognitive Impairment via Modulating Sphingolipid Metabolism and Gut Microbiota

Minghui Wang[a], Liuying Zhu[c], Jinqiang Liao[a], Lulu Bao [a], Hongyan Li[ab], Zeyuan Deng[ab], Jing Li[ab], Liufeng Zheng[ab], Bing Zhang[ab]*

[a]State Key Laboratory of Food Science and Resources, Nanchang University, Nanchang 330047, Jiangxi, China

[b]International Institute of Food Innovation, Nanchang University, Nanchang 330051, Jiangxi, China

[c]Ningbo Municipal Hospital of Traditional Chinese Medicine (TCM), Affiliated Hospital of Zhejiang Chinese Medical University, Ningbo, China.315010

*Corresponding authors:

*Phone/fax: +86-791-88304402, E-mail address: zhangbingair@126.com (Bing Zhang, PhD).

Table S1 Primer Information for qPCR

Target gene	forward primer	reverse primer
ACER2	TGTGGCATATTCTCATCTGCCT	CAATAAAAGCCCATTTCTCGCTG
SphK2	CACGGCGAGTTTGGTTCCTA	CTTCTGGCTTTGGGCGTAGT
S1PR1	ATGGTGTCCACTAGCATCCC	CGATGTTCAACTTGCCTGTGTAG
GAPDH	GAAGGTGAAGGTCGGAGTC	GAAGATGGTGATGGGATTTC

Fig S1. Spontaneous alternation in the Y-maze (A), total swimming distance (B), and average swimming speed (C) in the Morris water maze.

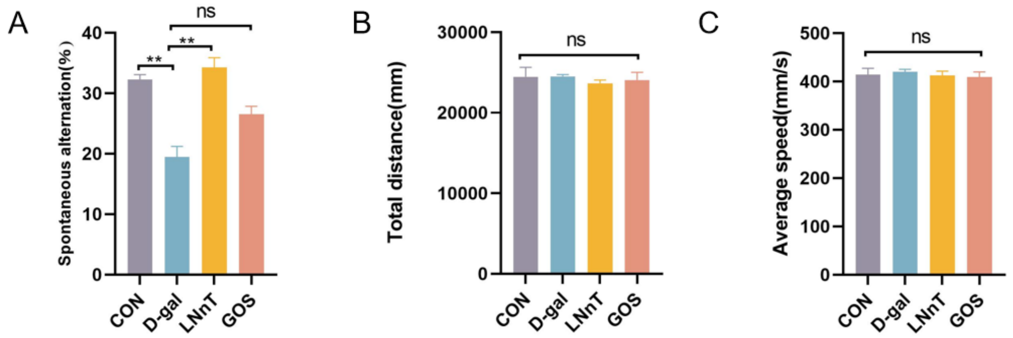

Fig S2. Venn diagram

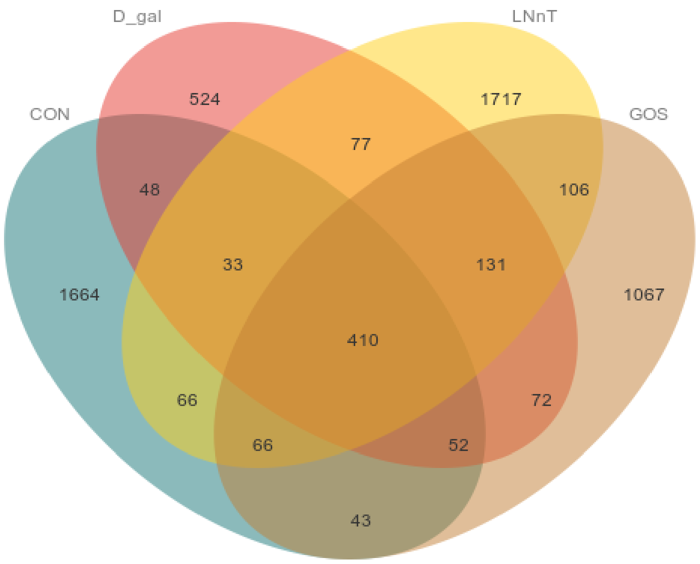

Fig S3. NMDS diagram.

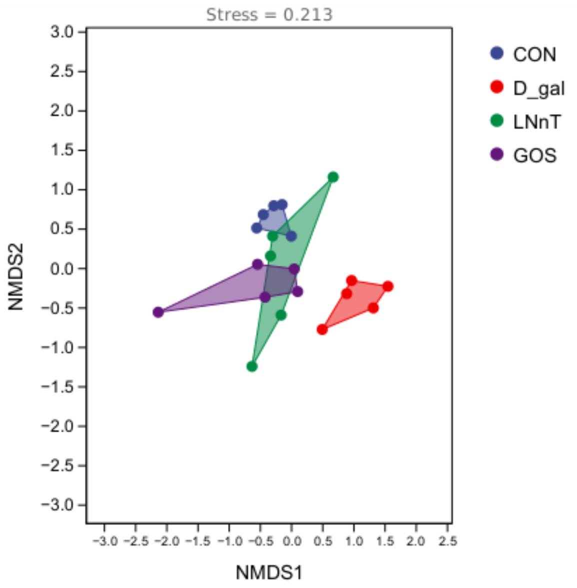

Fig S4. OPLS-DA score plots of metabolic profiles. (A、B) Control vs. D-gal group (R^2Y = 0.997, Q^2 = 0.974); (C、D) LNnT vs. D-gal group (R^2Y = 0.994, Q^2 = 0.954); (E、F) GOS vs. D-gal group (R^2Y = 0.988, Q^2 = 0.941).

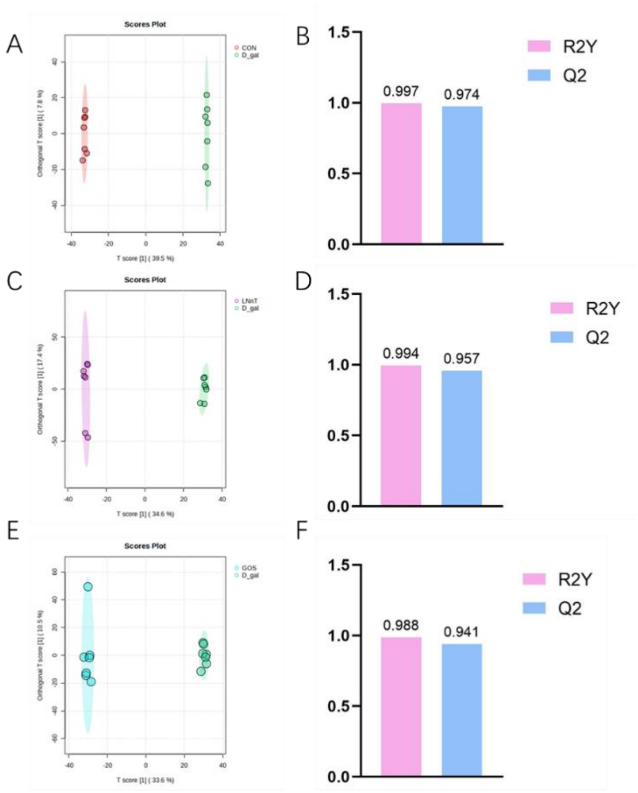

Supplement: Supplementary file 1 [file molecules-31-01410-s001.zip › molecules-4194590-supplementary.pdf]
